# Fault Diagnosis of Rotating Machinery Based on Improved Self-Supervised Learning Method and Very Few Labeled Samples

**DOI:** 10.3390/s22010192

**Published:** 2021-12-28

**Authors:** Meirong Wei, Yan Liu, Tao Zhang, Ze Wang, Jiaming Zhu

**Affiliations:** 1 School of Naval Architecture and Ocean Engineering, Huazhong University of Science and Technology, Wuhan 430074, China; meirongwei.eapp@gmail.com (M.W.); 15717322973@163.com (Z.W.); Jemicy_Chu@163.com (J.Z.); 2 Wuhan University Shenzhen Research Institute, Shenzhen 518057, China

**Keywords:** DTC-SimCLR, fault diagnosis with very few samples, data transformation, self-supervised learning

## Abstract

Convolution neural network (CNN)-based fault diagnosis methods have been widely adopted to obtain representative features and used to classify fault modes due to their prominent feature extraction capability. However, a large number of labeled samples are required to support the algorithm of CNNs, and, in the case of a limited amount of labeled samples, this may lead to overfitting. In this article, a novel ResNet-based method is developed to achieve fault diagnoses for machines with very few samples. To be specific, data transformation combinations (DTCs) are designed based on mutual information. It is worth noting that the selected DTC, which can complete the training process of the 1-D ResNet quickly without increasing the amount of training data, can be randomly used for any batch training data. Meanwhile, a self-supervised learning method called 1-D SimCLR is adopted to obtain an effective feature encoder, which can be optimized with very few unlabeled samples. Then, a fault diagnosis model named DTC-SimCLR is constructed by combining the selected data transformation combination, the obtained feature encoder and a fully-connected layer-based classifier. In DTC-SimCLR, the parameters of the feature encoder are fixed, and the classifier is trained with very few labeled samples. Two machine fault datasets from a cutting tooth and a bearing are conducted to evaluate the performance of DTC-SimCLR. Testing results show that DTC-SimCLR has superior performance and diagnostic accuracy with very few samples.

## 1. Introduction

Rotating machines, such as wind turbines, milling machines and turning lathes, are widely used in industrial applications [1,2,3]. For these kinds of machines, their reliability has a profound impact on operational safety and economic benefits. Consequently, it is crucial to perform intelligent fault diagnosis investigations for the machines [4,5,6,7].

In general, an intelligent fault diagnosis method mainly consists of condition data collection, feature extraction and fault classification. Among them, the feature extraction and the fault classification are of great significance to the diagnostic performance of an intelligent model.

For feature extraction, the main objective is to obtain the representative features, which is conducive to subsequent fault classification. The time domain, frequency domain and time–frequency domain have been widely used to extract the representative features from monitored signals. Zhang et al. [8] presented a gearbox fault diagnosis based on Fourier transform. Using the obtained time–frequency characteristics, the fault classification was realized. Pan et al. [9] utilized a time–frequency method SGMD to decompose the time series signal into a set of independent mode components for rotating machinery fault diagnosis. Li et al. [10] showed that a mixed matrix estimation model combined with the variational mode decomposition offered more effective features for the next fault classification of rotating machinery. In addition, other time–frequency methods, including local characteristic-scale decomposition [11], singular spectrum analysis [12], wavelet packet transform [13] and wavelet transform (WT) [14], have commonly been investigated in the field of fault diagnosis. Cheng et al. [15] used 74 wavelet basis functions to extract the time–frequency features of vibration signals, the quality index indicator was used to select the candidate wavelet basis function. From the experimental result, it was proved that the WT has a good ability to extract the fault features for classification. Ravikumar et al. [16] also applied the discrete WT to obtain the features from the vibration signals of an engine gearbox for fault classification. To sum up, advanced feature extraction methods applied to machine fault diagnosis are still the focus of research.

For fault classification, machine learning methods are usually applied to establish mapping relationships between extracted features and fault types. Machine learning methods, including support vector machine (SVM), logistic regression and k-nearest neighbor (KNN), have been commonly performed and investigated in machine fault diagnoses. Rudsari et al. [17] presented a modified SVM classifier to select the corresponding fault class for the extracted fault features. Gao et al. [18] adopted an efficient KNN-based method to identify the residual vector and judge the faulty sensor, in which different distance measures were analyzed and tested to optimize the final fault diagnosis performance. However, these machine learning methods provide a limited capability to establish the relationship between the extracted features and the fault modes, mainly including two limitations. One limitation of machine learning-based diagnostic models is the need to extract effective features based upon the support of expert experience or prior knowledge, which are obtained through tedious work. The other limitation is that training these methods generally requires a large amount of supervision information such as fault data. However, it is normally difficult or even impossible to collect fault data from the machinery in real application.

In order to overcome the limitations of feature extraction, deep learning-based fault diagnosis methods have attracted much attention. Feature extraction and fault classification can be performed simultaneously without any prior knowledge or expert experience by employing deep neural networks such as a convolutional neural network (CNN) [19]. Jin et al. [20] proposed a CNN-based model named LiNet, which can perform the feature extraction and the fault diagnosis at the same time. Ye et al. [21] demonstrated a deep morphological convolutional network that can learn the representative features from gearbox vibration signals and achieve excellent diagnostic performance. However, limitations still exist for these state-of-the-art diagnostic methods, especially when the machine cannot provide a large amount of fault data. Thus, the fault diagnosis of machines with very few labeled samples has become a key technology that is urgently in need of breakthroughs in actual industrial applications.

Up to now, weakly supervised learning [22], semi-supervised learning [23], active learning [24], imbalance learning [25], transfer learning [26,27] and metal learning [28] have been researched deeply and widely to solve problems. The semi-supervised learning and the transfer learning approaches have been widely applied in machine fault diagnoses. For example, Guo et al. [29] presented a semi-supervised fault diagnosis method based on a generative adversarial network (GAN). Specifically, GAN was used to generate fake fault data based on real fault data, then the generated fault data and the real data were used to optimize a fault classifier [30]. Dong et al. [31] used the diagnosis knowledge from massive and various simulation data to real scenario data through transfer learning where the fault data of the rolling element bearing is limited. Ruan et al. [32] carried out a relation-based semi-supervised method to reduce distribution discrepancy between the labeled and unlabeled samples. Kumar et al. [33] proposed a cross-entropy function that introduces sparsity in the CNN and creates an effective deep learning that can work in a situation when training data is not available in any abundance. Dixit et al. [34] presented a novel conditional auxiliary classifier GAN framework coupled with model agnostic metal learning and achieved high accuracy. In short, these methods are mainly aimed at increasing the fault data or reducing the demand for training data to solve the overfitting problem of the adopted deep learning model.

To address the overfitting problem caused by very few fault samples, a designed data transformation combination (DTC) is innovatively introduced into a constructed CNN model. In the DTC, the original signal samples are directly used as the training samples for 1-D CNN. Specifically, the original signal sample is firstly reshaped into a 2-D matrix, the data augmentations are randomly applied into this matrix to form the positive and negative sample. Meanwhile, a self-supervised learning method called SimCLR is constructed to obtain a 1-D ResNet-based feature encoder using very few samples. It is worth noting that its training samples are from the original signals, and the training complexity and the computer burden will not be increased. Then, a fault diagnosis model is established based on the obtained feature encoder and a fully-connected layer classifier. For this model, the parameters of the obtained feature encoder are fixed, and the classifier is optimized using very few fault samples.

The main contributions of this article are summarized as follows. Firstly, a DTC is designed and selected based on mutual information between the original signal sample and its corresponding transformed sample. Compared with traditional data augmentation technologies, the designed DTC does not increase the number of training samples and the original signal samples can be directly used by a 1-D CNN-based model, making its training effective. Moreover, by introducing the DTC, the complexity of the labeled samples is increased, and the overfitting risk of the CNN model can be alleviated. Secondly, a new self-supervised learning approach named 1-D SimCLR is constructed and adopted to obtain a 1-D ResNet-based feature encoder using few unlabeled fault samples. This feature encoder provides an effective representative feature for the subsequent fault classification task. Finally, a superior fault diagnosis model DTC-SimCLR is established based on the DTC, feature encoder and a fully-connected layer-based classifier, and the model can be trained using very few labeled fault samples. From the testing results, it can be seen that the proposed method achieves superior diagnostic performance compared with other existing methods.

The rest of this article is organized as follows. In Section 2, the framework of DTC-SimCLR is introduced. In Section 3, the details of DTC-SimCLR based on data transformations and SimCLR are presented. In Section 4, two datasets, including fault datasets from a cutting tooth and a bearing, are used to evaluate the effectiveness of DTC-SimCLR. Section 5 gives the conclusion.

## 2. The Framework of DTC-SimCLR Method

**Figure 1:**
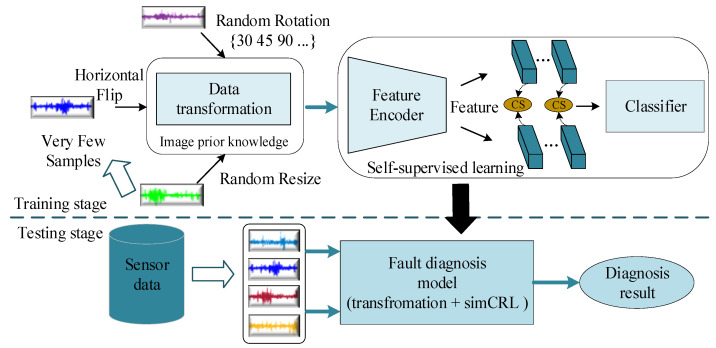
The framework for the machine fault diagnosis with very few samples.

The flowchart of the fault diagnosis method based on DTC-SimCLR, which includes two stages, namely the offline training stage and online testing stage, is presented in Figure 1.

In the offline training stage, condition data is collected from the sensors installed on the machine. In the designed DTC, all condition data are sampled into 2-D samples by a signal to sample method [35]. Data augmentations are used to increase the complexity of the samples through horizontal flip, random resize, rotation or combinations of these methods, etc. Subsequently, the transformed sample is flattened into the 1-D sample. It is worth noting that data augmentation is used as a natural step in the model training process, which does not increase the amount of training data and uses less data and faster training compared to GAN-based fault diagnosis methods. Then, few unlabeled signal samples are input into the proposed 1-D SimCLR to train a 1-D ResNet-based feature encoder. Subsequently, an effective representation for each signal sample is obtained. Finally, the obtained feature encoder is combined with a fully-connected layer-based classifier to establish a desired fault diagnosis model. In the optimization process of this diagnostic model, only the parameters of the classifier are modified using very few labeled signal samples.

In the online testing stage, real time data are collected with monitoring sensors as the training samples. The representation of the online sample is extracted using the feature encoder. Finally, the representation is identified by the classifier so as to output a series of alternative fault modes, which serve as the final diagnostic result.

## 3. The Fault Diagnosis Based on DTC-SimCLR under Few Labeled Samples

The fault diagnosis based on the proposed DTC-SimCLR method mainly includes the selection of data transformation, self-supervised learning of the feature encoder and fault classification.

### 3.1. Data Transformation for Signal Data Sample

Overfitting is one of the most common problems of deep learning models with very few training samples since the network training task becomes so simple that the trained network can easily lead to the preference of the network [36]. To overcome the overfitting problem, data augmentation is used to improve the complexity of training samples, which can increase the difficulty of the training task. Specifically, the data augmentation such as normalization, rotation, horizontal flip, gray scale, cropping, color jitter, affine, etc., are randomly applied to train samples. Herein, the parameters of each data transformation are determined by mutual information (MI) between two samples.

In the designed DTC, the samples of the machine’s vibration signal are converted into data samples. Assuming $X\;:=\left\{x^{\left(i\right)}\;\in \;X\right\}$ and $Z\;:=\left\{z^{\left(i\right)}\;\in \;Z\right\}$ are the original data samples and the transformed data samples, respectively, ${ℱ}_{\theta }\;:\;X\to \;Z$ denotes the transformations, where X and Z are the pixel of the original samples and the transformed samples, respectively. It is preferable to find proper parameters of different transformations ${ℱ}_{\theta }$ by maximizing the following objectives

(1) $${\left(ℱ,\hat{\theta }\;\right)}_{G}=argmaxℳ{ℐ}_{{ℱ}_{\theta }}\left(X;\;Z\right)$$

where $ℳ{ℐ}_{{ℱ}_{\theta }}$ is MI calculation provided by different transformations with parameter θ. $Z={ℱ}_{\theta }\left(X\right)$ denotes the output samples.

A signal data sample is sampled from the raw condition signal of rotating machinery. It can be described as the following

(2) $$x^{m\times n}=reshape\left(s^{mn}\right)$$

where a condition signal is sampled with a data sample $s^{mn}$ length of mn, and then it is adjusted to a length of m and width of n using a reshaping operation to lift the two-dimensional data.

Then, given the data sample $x\in {\mathbb{R}}^{m\times n}$ of size m×n, the data transformation can be defined as

(3) $${\tilde{x}}_{ij}=TP\left(x_{ij}\right)$$

where TP denotes the transformation operation, $x_{ij}$ is the original data sample and ${\tilde{x}}_{ij}$ is the transformed data sample.

Finally, the obtained 2-D matrix is flattened

(4) $$x=F\left({\tilde{x}}_{ij},axis=-1\right)$$

**Figure 2:**
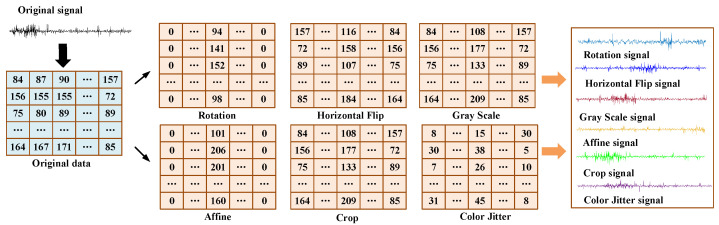
The data transformation results of an original data sample.

An original sample and several transformed samples through different data transformations are plotted in Figure 2. It is found that the data point of each transformed data is different from the original data. Thus, the training task will become complex and the overfitting risk may be alleviated. It is to be noted that only a part of the randomly selected training samples is transformed in the training process. The training samples and computing burden will not be increased, and the training process can be quickly completed.

### 3.2. The Self-Supervised Learning of Representations Based on SimCLR

**Figure 3:**
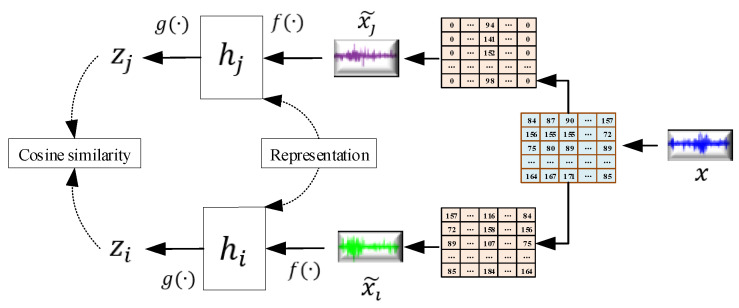
The process of the self-supervised learning of SimCLR.

Self-supervised learning of the SimCLR mainly includes four steps, namely data transformation, feature extraction, feature nonlinear mapping and contrastive loss function construction [37]. The process of self-supervised learning of the proposed 1-D SimCLR for the appropriate representation z is shown in Figure 3. In the data transformation step, the aforementioned data transformations are analyzed and combined. Specifically, each data sample x is converted into two correlated views using a selected DTC, denoted with $\tilde{x_{i}}$ and $\tilde{x_{j}}$, which are a positive pair, and the pairs of other data samples are negative pairs. As shown in Section 4, different DTCs were performed and tested, among which, two transformations, namely the normalization and the combination of normalization, rotation and cropping, achieved good performance in two case studies.

In the feature extraction step, a feature encoder f(·) is used to extract the representative features from the transformed sample after the positive pairs and the negative pairs are obtained. In this article, the ResNet is applied to obtain the representation as follows

(5) $$h_{i}=f\left({\tilde{x}}_{i}\right)=ResNet\left({\tilde{x}}_{i}\right)$$

where the $h_{i}$ is the output of the last average pooling layer.

In the feature nonlinear mapping step, a fully-connected neural network g(·) is adopted to nonlinearly fit the extracted representation of the output of f(·). Here, the mapping relationship can be expressed as

(6) $$z_{i}=W^{1}\sigma \left(W^{2}h_{i}\right)$$

where σ is the ReLu activate function, and $W^{1}$ and $W^{2}$ is the bias. From the description of the original SimCLR, this $z_{i}$ is more beneficial to obtain the contractive loss than $h_{i}$.

In the last step, a set of positive pairs $\left\{{\tilde{x}}_{k}\right\}$ including $\tilde{x_{i}}$ and $\tilde{x_{j}}$ is given, and a classification task aims to classify $\tilde{x_{j}}$ and $\tilde{x_{i}}$ in ${\left\{{\tilde{x}}_{k}\right\}}_{k\neq i}$. A minibatch of N training samples are sampled, thus, 2N samples can be derived. Then, one positive pair is selected, and other 2(N − 1) samples within a minibatch are treated as the negative samples. Let cosine similarity denote the distance between the positive sample u and the negative sample v. This process is described as follows

(7) $$sim\left(u,v\right)=\frac{u^{T}v}{\left||u||||v|\right|}$$

Then, the contractive loss function for the positive pair of samples (i,j) can be defined as follows

(8) $$Loss_{i,j}=-\log\frac{\exp\left(sim\left(z_{i},z_{j}\right)/\tau \right)}{\sum_{k=1,k\neq i}^{2N}\exp\left(sim\left(z_{i},z_{k}\right)/\tau \right)}$$

where τ denotes the temperature parameter.

In the above constructed loss function, all positive pairs including (i,j) and (j,i) are computed in each minibatch. Based on the contractive loss function, the parameters of f(·) and g(·) are optimized through the stochastic gradient descent algorithm. Then, a useful feature encoder can be obtained to form the representation for x.

### 3.3. Fault Diagnosis under Very Few Fault Samples

To implement the fault diagnosis of a machine, a novel model is proposed based on the selected DTC and the obtained feature encoder. It aims to establish the mapping relationship between the extracted representations and the fault types. Once the self-supervised learning process of the encoder has been completed, a classifier based on a fully-connected layer is combined with the obtained encoder to establish the final fault diagnosis model. The output $z_{i}$ of the encoder for the i-*th* input data $x_{i}$ is defined by

(9) $$z_{i}=g\left(f\left(x_{i}\right)\right)=W^{1}\sigma \;\left(W^{2}f\left(x_{i}\right)\right)$$

(10) $$f_{c}\left(x_{i}\right)=y_{i}=\sigma \left(W^{T}*x_{i}+b\right)$$

(11) $$f_{p}\left(y_{i}\right)=y_{i}^{\prime }=\max\left\{y_{i}\right\}$$

where $f_{c}\left(\cdot \right)$ is the convolutional operation and $f_{p}\left(\cdot \right)$ is the pooling operation. f(·) denotes the group of $f_{c}\left(\cdot \right)$ and $f_{p}\left(\cdot \right)$. g(·) is a fully-connected layer.

Then, the representation obtained from the feature encoder is fed into the next two additional layers. The first fully-connected layer with ReLu activate function is used for the nonlinear fitting representation. The classification layer with the Softmax units gives a conditional probability for each fault mode.

For instance, there are n fault modes of machine conditions in total for the input sample x, and the output probability $O_{j}\in \left[0,1\right]$ for fault mode j is calculated as

(12) $$O_{j}=\frac{e^{\left(\theta^{\left(j\right)}x\right)}}{\sum_{j=1}^{n}e^{\left(\theta^{\left(j\right)}x\right)}},j=1,2,\cdots n$$

(13) $$\sum_{j=1}^{n}O_{j}=1$$

where $\theta^{\left(j\right)}$ denotes the parameters of the encoder and the constructed classifier. It is worth noting that the parameters of the encoder are obtained from the self-supervised learning process, and the parameters of the classifier are optimized using very few labeled samples.

## 4. Case Studies and Experiments Results

To evaluate the performance of DTC-SimCLR with very few samples, two fault datasets from the cutting tooth of a milling machine and the bearing of a rotating machine are investigated individually. These two cases are studied on an Ubuntu system platform with a 48 Intel Core 2.2 GHz processor and 256 GB RAM. NVIDIA GeForce RTX 2080Ti is used to accelerate the graphic calculations. All algorithms are developed using python code on Pytorch, where most of them are original.

### 4.1. Case One: Cutting Tooth Fault Diagnosis

#### 4.1.1. Experimental Setup and Data Description

**Figure 4:**
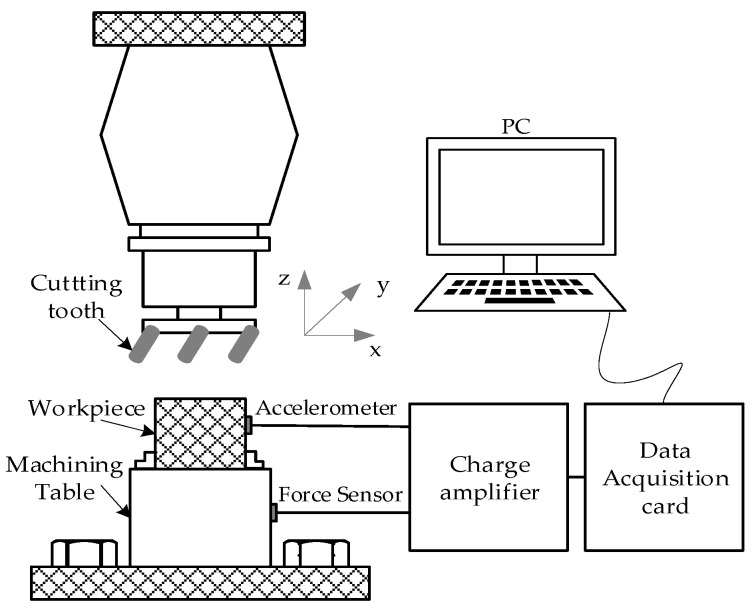
The experimental setup for a cutting tool.

A dataset of vibration signals from the cutting tooth of a high-speed CNC milling machine was collected under different force conditions [38,39]. Figure 4 shows the schematic diagram of the experimental platform. The spindle speed of the cutter was 10,400 RPM. Feed rate was 1555 mm/min. The Y depth of cut (radial) was 0.125 mm, and the Z depth of cut (axial) was 0.2 mm. To acquire online data related to this CNC machine’s operation condition, a Kistler quartz 3-component platform dynamometer was mounted between the workpiece and the machining table to measure cutting forces, while three Kistler Piezo accelerometers were mounted on the workpiece to measure the machine tool vibration in *x*-, *y*- and *z*-directions, respectively. In-process measurements including force and vibration in three directions (*x*, *y*, *z*) were sampled with a continuous frequency of 50 KHz during the tool wear test. Therefore, the sampled data consist of seven channels: force in three directions, vibration in three directions and AE-rms. According to the degree of wear depth measured with a LEICA MZ12 microscope, the conditions of the cutting tooth are divided into five fault stages, namely initial wear (IW), mild wear (MW), rapid wear (RW), severe wear (SW) and complete wear (CW). Table 1 lists the wear stages division results. These wear stages can be regarded as different fault types for the milling machine.

In the experiment, there were six individual cutter records, c1…c6. Each record has 315 vibration signal curves, and each signal curve contains 222,889 data points. In this article, the cutter tooth record c4 was selected to evaluate the performance of the DTC-SimCLR method.

Table 2 presents the details of the fault dataset. For each cutting tooth condition, 1000 samples were collected from its corresponding vibration signals through a sliding window with a length of 1024 data points. In order to evaluate the effectiveness of the proposed fault diagnosis method under very few samples, five datasets with 1% of total samples per dataset (10 samples/dataset) were selected randomly as labeled samples. Three hundred samples were used as the testing samples. In this article, each signal sample was reshaped into a 32 × 32 data sample.

#### 4.1.2. Results and Discussion

**Figure 5:**
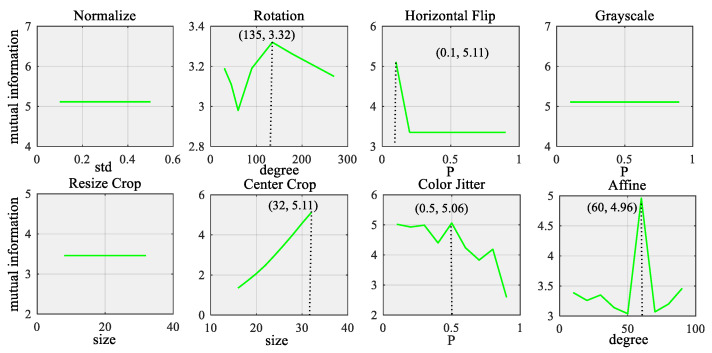
The mutual information of the original data samples under different transformations.

In this case study, the fault diagnosis problem of the machine is naturally a multi-class identification task that includes five classes. It aims to perform the fault diagnosis of the cutting tooth with very few labeled samples (50 samples in total). Specifically, different data transformations, such as normalization, rotation, horizontal flip, grayscale, resize crop, center cropping, color jitter and affine, are discussed. The parameters of these transformations are selected according to the MI between the original data sample and their corresponding transformed samples. Figure 5 shows the process of determining the optimal parameters for different transformations using the maximum mutual information of the original data and the transformed data. It was found that the with rotation with its degree equaling 135, horizontal flip with its probability equaling 0.1, center cropping with its size equaling 32 × 32, color jitter with its probability equaling 0.5, affine with its degree equaling 60 the maximum MI value was reached.

The parameters of the DTC-SimCLR are listed in Table 3. In this article, the ResNet18 with 16 major convolutional layers in total is introduced as the feature encoder. The output feature dimension is 128. More details can be referred to in [40]. The training epoch of the proposed 1-D SimCLR is set to 200. After the training epoch has been reached, the feature encoder is fixed and used for the next fault diagnosis scenario.

Then, a fault diagnosis model based on DTC-SimCLR is constructed. The parameters of the classifier for the fully-connected layer with ReLU and the classification layer with the Softmax are 256 and 5, respectively. This model is optimized with very few labeled samples. Further evaluation and analysis are discussed in the following scenarios.

(1) The comparison of DTCs

The accuracy of models treated by different DTCs were tested and the results are presented in Table 4. One trial was carried out to diagnose five conditions of a cutting tooth. The result shows that the output performance reaches the accuracy of 93.4% when the input data samples are processed with normalization alone, better than other DTCs.

(2) CNN without DTC vs. CNN with DTC vs. DTC-SimCLR

**Figure 6:**
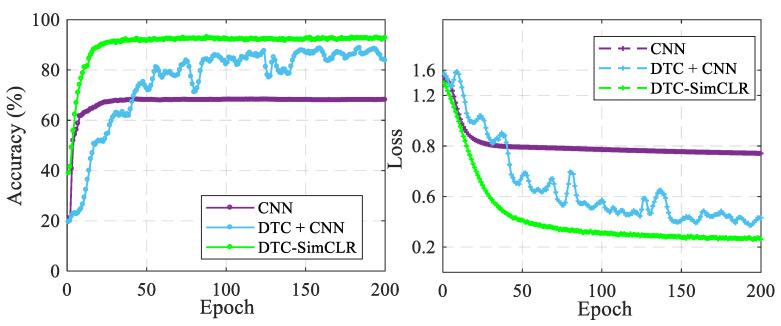
The testing accuracy and testing loss comparison results between the CNN and the proposed methods.

Three diagnosis models, CNN without DTC, CNN with DTC and DTC-SimCLR were trained to demonstrate their performance, and the training results are shown in Figure 6. Compared to CNN without DTC, it is clear that CNN with DTC can reach a higher testing accuracy and a lower testing loss but requires more epochs to become stable. Meanwhile, DTC-SimCLR demonstrates even better diagnosis performance than the other two. It can attractively achieve the highest accuracy and the lowest loss with few epochs among these three methods.

**Figure 7:**
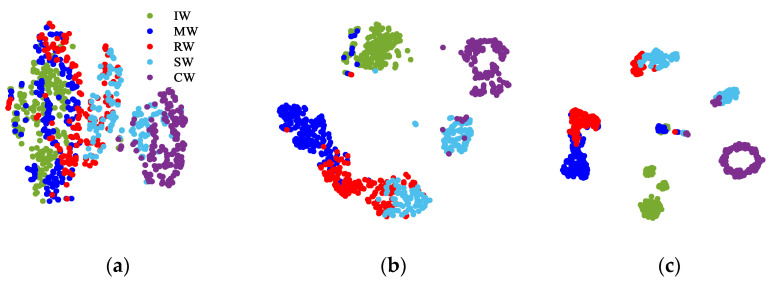
The visualization of the learned features by different methods. (**a**)CNN without DTC; (**b**) CNN with DTC; (**c**) DTC-SimCLR.

To show the advancement of DTC-SimCLR in extracting representations, a t-SNE technique was used to reduce the dimension of the representations into two dimensions (2D) for visualization. The representations were extracted from the testing samples using the feature encoder. Figure 7 plots the 2D representations of the testing samples, where these points with five colors denote five cutting tooth conditions. From Figure 7a,b, it can be found that CNN with DTC can enhance cluster performance of the extracted representations. Furthermore, from Figure 7c, it is found that DTC-SimCLR can make the extracted representation much more separable. This result illustrates that incorporating the normalization transformation with the SimCLR can greatly improve feature extracting performance and, therefore, fault diagnosis capability.

**Figure 8:**
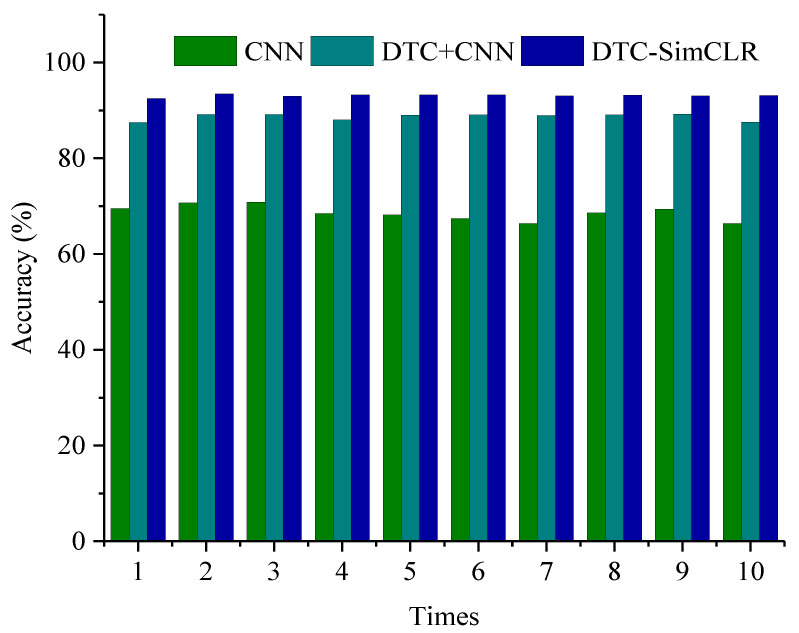
The ten trial results among different methods.

In addition, ten more trials were carried out to demonstrate the performances of the three above models and the results are compared as shown in Figure 8. In each trial, DTC-SimCLR shows a higher testing accuracy than the other two. It illustrates that the proposed method can achieve stable and excellent fault diagnosis performance.

Table 5 gives the test accuracy of the fault diagnosis models obtained using different numbers of labeled samples. Here, three models trained from three datasets are compared, which consist of 10 samples, 5 samples and 1 sample per fault category, respectively. The comparative results of the final model performance show that the models trained using the dataset of 10 labeled samples per fault type are more satisfactory.

(3) DTC-SimCLR vs. other common methods.

To further demonstrate the advantage of this proposed method, Complex tree, Cubic SVM with cubic kernel, Ensemble KNN and Weighted KNN, were used to compare with the proposed DTC-SimCLR method. Here, the samples for these models are the original signal samples without reshaping operation. The testing samples are used to evaluate their performance. Table 6 displays the testing accuracy and training time of the models under different sizes of training samples. The average testing accuracy of the proposed DTC-SimCLR method is 93.1% under 50 samples, which is obviously higher than other models. The performance of the proposed method is close to the diagnostic performance of the CNN trained with 3500 labeled samples, whose accuracy is 93.67%. This result shows the proposed DTC-SimCLR method can overcome the limitation of machine fault diagnosis when there are very few labeled samples. Moreover, a method based on WT [41,42] and Cubic SVM is also presented in Table 6. The WT with Daubechies function is used to extract time–frequency features of the original signal samples, and then the Cubic SVM is used to classify them. From the test result, it can be seen that the proposed method has good end-to-end fault diagnosis capability.

### 4.2. Case Two: Bearing Fault Diagnosis

#### 4.2.1. Dataset Description

**Figure 9:**
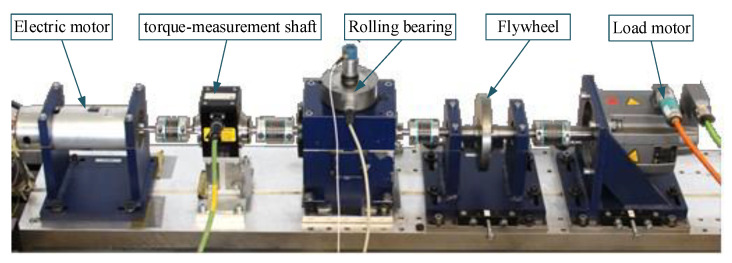
Experimental setup of the bearing dataset.

In this case, the dataset of the bearing condition was collected from the test rig designed at the Chair of Design and Drive Technology, Paderborn University [43]. The test rig is shown in Figure 9, which mainly consists of an electric motor, torque-measurement shaft, rolling bearing test module, flywheel and load motor. The piezoelectric accelerometer (Model No. 336c04, PCB Piezotronics, Inc.) and a charge amplifier (Type 5015A, Kistler Group) mounted on the top end of the rolling bearing test module are used to measure the condition signals of the testing bearings with a sampling rate of 64 KHz. Three bearing conditions, including normal condition, inner race fault (IRF) condition and outer race fault (ORF) condition, were analyzed. Each bearing condition contained the signals from the five testing bearings. Fifteen bearing datasets were tested where each bearing dataset contained ten segments and the sampling time of each segment was 4 s. There were 100 segments with 256,000 data points for each bearing condition. In total, 3000 samples were collected from the bearing condition signals where the data samples were collected through a sliding window with a length of 1024 data points. Table 7 describes the details of the constructed bearing dataset.

Similarly, three datasets with 1% of total samples per dataset (10 samples/dataset) were selected as the labeled samples, 300 samples per bearing condition were used as the testing samples. All samples were uniformly converted into 2D data samples. The size of the samples was also 32 × 32.

#### 4.2.2. Results and Discussion

(1) The comparison of DTCs

**Figure 10:**
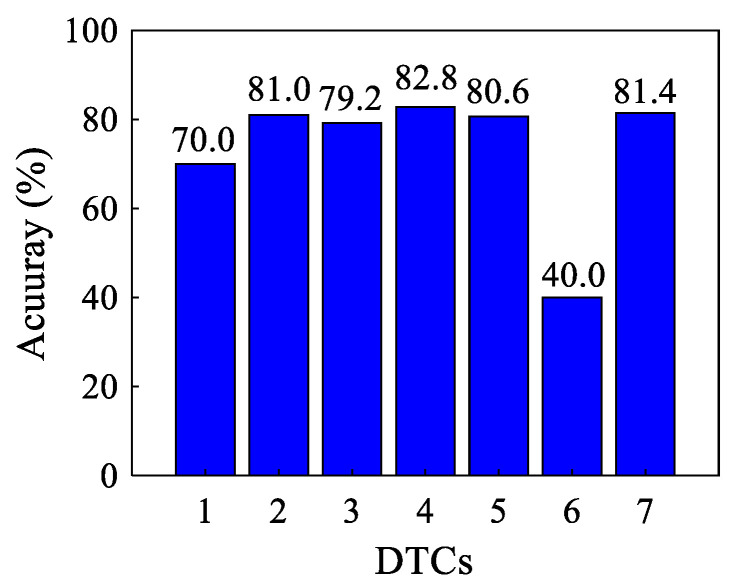
The testing performance comparison under different transformation combinations.

A proper transformation combination is first selected. Figure 10 shows the testing accuracy of the proposed DTC-SimCLR method under different transformation combinations as listed in Table 4. The details of these combinations are presented in Figure 5. The feature encoder is constructed by using the parameters, which are described in case one, and the feature encoder is optimized by using DTC-SimCLR presented in Table 4.

(2) CNN without DTC vs. CNN with DTC vs. DTC-SimCLR

**Figure 11:**
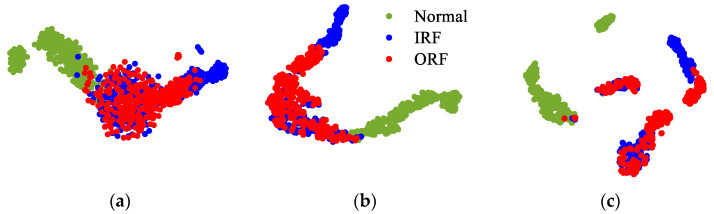
The testing performance of the proposed transform among different transform combinations. (**a**) CNN without DTC; (**b**) CNN with DTC; (**c**) DTC-SimCLR.

Figure 11 plots the 2D visualizations for the high-level representations of the testing samples. Here, the extracted 2D representations by CNN without DTC are shown in Figure 11a, CNN with DTC is applied in Figure 11b and the visualization result of the proposed DTC-SimCLR method is presented in Figure 11c. From the separability of the results, it is clear that the proposed DTC-SimCLR method can learn more effective information and good representation from bearing conditions signals.

Table 8 lists ten more trial results from different methods. The average testing accuracy of CNN without DTC is 67.32%, CNN with DTC is 79.24% and DTC-SimCLR is 83.52%. It is found that selected DTC has the capability to improve diagnostic performance. Moreover, from the testing accuracy of the proposed DTC-SimCLR method, it shows that SimCLR can improve the capability to learn useful representations compared to CNN.

(3) DTC-SimCLR vs. other common methods.

Table 9 gives the comparison results from the existing popular methods under different labeled samples. When the labeled samples are very few, the proposed DTC-SimCLR method still has satisfactory diagnostic performance, and it is much better than other methods. Furthermore, the testing accuracy of the proposed method trained with 30 labeled samples is significantly higher than other common machine learning methods, and it is close to the testing performance of the CNN trained with 2100 labeled samples. It is found that the proposed method has the potential ability to surpass the diagnostic model trained with a large number of labeled samples.

## 5. Conclusions

This article has proposed a novel fault diagnosis DTC-SimCLR method for rotating machinery based on the designed transformation combination (DTC) with the developed 1-D SimCLR. In this method, an appropriate DTC is selected and used to make the training sample more complex, and alleviates the overfitting problem when the training samples are limited. The developed 1-D SimCLR is applied to optimize the constructed 1-D CNN-based feature encoder for extracting the effective fault feature from original signal samples. Then, the obtained feature encoder and the fully-connected layer-based classifier are combined as the desired fault diagnosis model, which can be finely trained with a very small number of labeled fault samples. Unlike other traditional deep learning methods, which require many labeled fault samples to implement parameter training, the proposed method can achieve the training process of a diagnosis model with very few fault samples. From the experimental results, it is found that the proposed method has satisfactory training performance and superior diagnostic accuracy compared with other existing methods when only a few fault samples can be reached. In future work, the proposed DCT-SimCLR method has the potential to achieve transfer learning between different machine objects because it only requires a few labeled samples.

## Figures and Tables

**Table 1:** The Wear Stage Division Results.

Wear Stage	Initial Wear	Smooth Wear	Rapid Wear	Severe Wear	Complete Wear
VB	0~94.5	94.5~113.0	113~134.2	134.2~165	>165.0
Signal	1~95	95~175	176~240	241~297	298~315

**Table 2:** The Description of The Cutting Tooth Fault Dataset.

Fault	Label	Condition Samples	Labeled Samples	Testing Samples
IW	1	1000	10	300
MW	2	1000	10	300
RW	3	1000	10	300
SW	4	1000	10	300
CW	5	1000	10	300

**Table 3:** The Parameters of the proposed 1-D SimCLR.

Parameters	Size
Input size	1024
Temperature (τ)	10
Feature encoder	16 convolutional layers
Output size	128
Training epoch	200

**Table 4:** The Testing Performance Comparison Under Different Transformation Combinations.

No.	1	2	3	4	5	6	7	8	Accuracy
1	Normalization	Rotation	horizontal flip	Grayscale	Color Jitter	Affine	Resize crop	Center Crop	28.31%
2	Normalization	Rotation	horizontal flip	Grayscale	-	-	Resize crop	-	83.50%
3	Normalization	Rotation	horizontal flip	-	-	-	Resize crop	-	80.24%
4	Normalization	Rotation	-	-	-	-	Resize crop	-	81.44%
5	Normalization	-	-	-	-	-	Resize crop	-	90.24%
6	-	Rotation	-	-	-	-	-	-	20.32%
7	Normalization	-	-	-	-	-	-	-	93.40%

**Table 5:** The Comparison Results Among Different Methods and DTC-SimCLR.

Method	Mean Testing Accuracy ± std (%)		
	50 Samples	25 Samples	5 Samples
DTC-SimCLR	93.11 ±0.24	75.39 ± 2.19	30.70 ±5.76

**Table 6:** The Comparison Results Among Different Methods and DTC-SimCLR.

Methods	Testing Accuracy (%)		Training Time (s)	
	50 Labeled Samples	3500 Labeled Samples	50 Labeled Samples	3500 Labeled Samples
Complex tree	28.9	59.3	5.68	108.16
Cubic SVM	47.9	92.1	8.24	215.30
Ensemble KNN	50.4	81.0	14.42	1695.9
Weighted KNN	47.9	81.6	10.27	724.81
WT + Cubic SVM	39.3	66.7	4.55	724.32
CNN	68.6	93.7	18.52	772.59
GAN	85.6	-	500.23	-
DTC-SimCLR	93.1	-	19.10	-

**Table 7:** Bearing Dataset Details.

Conditions	Label	Condition Samples	Labeled Samples	Testing Samples
Normal	1	1000	10	300
IRF	2	1000	10	300
ORF	3	1000	10	300

**Table 8:** The Comparison Results Among Different Methods and DTC-SimCLR (%).

Method	1	2	3	4	5	6	7	8	9	10	Average
CNN	67.1	67.0	66.1	65.8	68.7	69.9	66.0	67.7	67.6	67.3	67.32
DTC + CNN	79.7	77.3	79.9	79.9	79.3	79.2	79.8	78.3	79.8	79.2	79.24
DTC-SimCLR	82.0	85.8	82.2	83.4	83.8	82.8	83.4	82.6	85.2	84.0	83.52

**Table 9:** The Comparison Results Among Different Methods and DTC-SimCLR.

Methods	Testing Accuracy (%)		Training Time (s)	
	30 Labeled Samples	2100 Labeled Samples	30 Labeled Samples	2100 Labeled Samples
Complex tree	46.7	56.0	2.44	13.39
Cubic SVM	43.3	63.9	3.02	24.42
Ensemble KNN	36.7	52.0	7.76	84.31
Cosine KNN	53.3	68.8	5.46	37.74
WT + Cubic SVM	34.5	63.6	2.35	56.56
CNN	67.32	84.00	8.32	182.51
GAN	71.2	-	326.5	-
DTC-SimCLR	83.52	-	8.54	-

## Data Availability

Publicly available datasets were analyzed in this study. One dataset can be found in 2010 PHM society conference data challenge: https://www.phmsociety.org/competition/phm/10 (accessed on 15 November 2021). The other one can be found at https://mb.uni-paderborn.de/en/kat/main-research/datacenter/bearing-datacenter/data-sets-and-download (accessed on 15 November 2021).
